# Immunotherapy for HER2-Positive Breast Cancer: Clinical Evidence and Future Perspectives

**DOI:** 10.3390/cancers14092136

**Published:** 2022-04-25

**Authors:** Elisa Agostinetto, Filippo Montemurro, Fabio Puglisi, Carmen Criscitiello, Giampaolo Bianchini, Lucia Del Mastro, Martino Introna, Carlo Tondini, Armando Santoro, Alberto Zambelli

**Affiliations:** 1 Academic Trials Promoting Team, Institut Jules Bordet, L’Université Libre de Bruxelles (U.L.B), 1070 Brussels, Belgium; elisa.agostinetto@bordet.be; 2 Department of Biomedical Sciences, Humanitas University, Via Rita Levi Montalcini 4, Pieve Emanuele, 20072 Milan, Italy; armando.santoro@cancercenter.humanitas.it; 3 IRCCS Humanitas Research Hospital, Humanitas Cancer Center, Via Manzoni 56, Rozzano, 20089 Milan, Italy; 4 Direzione Breast Unit, Candiolo Cancer Institute, FPO-IRCCS, 10060 Candiolo, Italy; filippo.montemurro@ircc.it; 5 Department of Medical Oncology, CRO Aviano, National Cancer Institute, IRCCS, 33081 Aviano, Italy; fabio.puglisi@uniud.it; 6 Department of Medicine (DAME), University of Udine, 33100 Udine, Italy; 7 Division of Early Drug Development, European Institute of Oncology IRCCS, 20141 Milan, Italy; carmen.criscitiello@ieo.it; 8 Department of Oncology and Hematology, University of Milan, 20122 Milan, Italy; 9 Department of Medical Oncology, IRCCS Ospedale San Raffaele, 20132 Milan, Italy; bianchini.giampaolo@hsr.it; 10 IRCCS Ospedale Policlinico San Martino, Clinica di Oncologia Medica, 16132 Genova, Italy; lucia.delmastro@unige.it; 11 Dipartimento di Medicina Interna e Specialità Medica, Università di Genova, 16124 Genova, Italy; 12 UOS Centro di Terapia Cellulare “G. Lanzani”, ASST Papa Giovanni XXIII, 24127 Bergamo, Italy; mintrona@asst-pg23.it; 13 Medical Oncology Unit, ASST Papa Giovanni XXIII, Piazza OMS 1, 27100 Bergamo, Italy; ctondini@asst-pg23.it

**Keywords:** HER2-positive breast cancer, immunotherapy, immune checkpoint inhibitors, antibody–drug conjugates, CAR-T cells, vaccines

## Abstract

**Simple Summary:**

Human epidermal growth factor receptor 2 (HER2) positive breast cancer is a heterogeneous disease. Among different therapeutic approaches, immunotherapies represent a relevant option for HER2-positive breast cancer patients, both in the adjuvant and metastatic setting. Starting from the dramatic clinical improvement observed with the advent of trastuzumab, which embodied a partly immune-mediated mechanism, a new wave of immunotherapies is now under investigation, including the novel HER2-directed monoclonal antibodies, the antibody–drug conjugates, the immune checkpoint inhibitors, the adoptive T-cell therapies, and therapeutic vaccines. In this narrative review, we present the most important clinical evidence on immunotherapy in HER2-positive breast cancer, and we critically discuss the uncertainties and opportunities in this evolving field of immune-oncology.

**Abstract:**

Breast cancer is the most common malignancy among women worldwide, and HER2-positive breast cancer accounts for approximately 15% of all breast cancer diagnoses. The advent of HER2-targeting therapies has dramatically improved the survival of these patients, significantly reducing their risk of recurrence and death. However, as a significant proportion of patients ultimately develop resistance to these therapies, it is extremely important to identify new treatments to further improve their clinical outcomes. Immunotherapy has revolutionized the treatment and history of several cancer types, and it has already been approved as a standard of care for patients with triple-negative breast cancer. Based on a strong preclinical rationale, immunotherapy in HER2-positive breast cancer represents an intriguing field that is currently under clinical investigation. There is a close interplay between HER2-targeting therapies (both approved and under investigation) and the immune system, and several new immunotherapeutic strategies, including immune checkpoint inhibitors, CAR-T cells and therapeutic vaccines, are being studied in this disease. In this narrative review, we discuss the clinical evidence and the future perspectives of immunotherapy for patients with HER2-positive breast cancer.

## 1. Introduction

Breast cancer is a heterogeneous disease, traditionally classified according to the expression of hormonal receptors and of human epidermal growth factor receptor 2 (HER2) [1]. HER2-positive breast cancer accounts for approximately 15% of all breast cancer diagnoses, and it is characterized by a poor prognosis in the absence of specific HER2-targeting therapies [2]. The advent of HER2-targeting therapies has dramatically improved the survival of these patients, significantly reducing their risk of recurrence and death [2,3]. However, as a significant proportion of patients ultimately develop resistance to these therapies, it is extremely important to identify new treatments to further improve their clinical outcomes.

Immunotherapy has revolutionized the treatment and history of several cancer types, becoming a well-established standard of care [4]. With its capability of inducing durable and prolonged responses, immunotherapy has also been studied in cancer types traditionally considered not immunogenic, such as breast cancer [5,6]. Among breast cancer subtypes, triple-negative breast cancer (TNBC) is considered the most immunogenic one [7,8,9,10]; hence, the only current approval of immune checkpoint inhibitors in breast cancer is for patients with TNBC [11]. However, the role of immunotherapy in breast cancer is also being explored in other populations and disease settings, including the HER2-positive subtype.

There is a strong rationale supporting the investigation of immunotherapy in HER2-positive breast cancer based on its high tumor mutational burden (TMB) and high levels of tumor-infiltrating lymphocytes (TILs) [8,9]. The median level of stromal TILs in HER2-positive tumors ranges between 15 and 20%, with differences according to hormone receptor status (i.e., median TILs level is lower in hormone receptor-positive/HER2-positive tumors than in hormone receptor-negative/HER2-positive tumors) [9]. High TILs in primary HER2-positive tumors correlate with increased pathological complete response (pCR) rates and improved disease-free survival (DFS) and overall survival (OS) [12].

In the present review, we aim to illustrate the current evidence of immunotherapy in HER2-positive breast cancer and to summarize data from early clinical investigations of promising agents that could proceed to future development.

## 2. Monoclonal Antibodies

**Figure 1:**
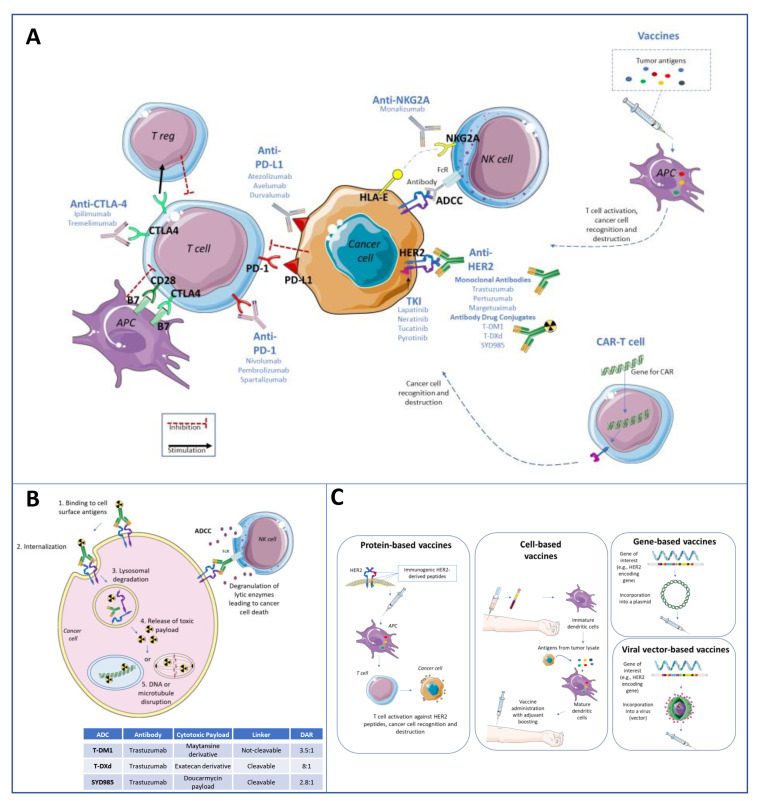
Simplified representation of immunotherapeutic strategies in HER2-positive breast cancer (**A**). Monoclonal antibodies (e.g., trastuzumab, pertuzumab) and antibody–drug conjugates (ADCs) (e.g., T-DM1, T-DXd, SYD985) exert both a direct anti-tumor effect, by blocking and modulating the HER2 signaling, but also an indirect effect, by activating the immune system through the so-called antibody-dependent cellular cytotoxicity (ADCC) (**B**). ADCC consists of the cellular lysis of tumor cells induced by immune effectors via an antibody targeting action that involves the FcR signaling. In other words, antibodies can recognize and bind to antigens expressed on the surface of tumor cells and create a “bridge” between the tumor cell and the immune effector cells expressing Fc-gamma receptors. The immune cell activated via the FcR signaling releases lytic enzymes (i.e., granzyme B, perforin) able to induce the apoptosis of the tumor cell. ADCs are composed of an anti-HER2 monoclonal antibody, bound via a linker (cleavable or not) to a cytotoxic agent. Hence, their anti-tumor properties consist not only in the blockade of the HER2 signaling pathway and ADCC induction, but also in the internalization of the cytotoxic agent by HER2 expressing cells, with a consequent more potent cytotoxic effect within tumor cells and less toxicity on healthy tissue. (**C**) reports a simplified representation of the therapeutic vaccines under development for patients with HER2-positive breast cancer (protein-based, cell-based, gene-based, and viral vector-based vaccines).

Anti-HER2 monoclonal antibodies (i.e., trastuzumab and pertuzumab) have a well-established role in the treatment of patients with HER2-positive breast cancer, both in the early [3,13,14] and advanced settings [15]. Nevertheless, more than two decades after their first introduction in the clinic, the mechanism of action of HER2-targeted monoclonal antibodies is still an area of active research. Indeed, although, for a long time, their clinical benefit has been exclusively attributed to the direct effect on HER2 and to the downregulation of its oncogenic intracellular pathway [16], in recent decades, it has become clear that monoclonal antibodies exert their action also through the activation of the immune system [17]. Thus, monoclonal antibodies exert both a direct anti-tumor effect by blocking and modulating the HER2 signaling and also an indirect effect by activating the immune system through the so-called antibody-dependent cellular cytotoxicity (ADCC) (Figure 1). ADCC consists of the cellular lysis of tumor cells induced by immune effectors via an antibody targeting action that involves the Fc receptor signaling. In other words, antibodies can recognize and bind to antigens expressed on the surface of tumor cells and create a “bridge” between the tumor cell and the immune effector cells expressing Fc-gamma receptors. The immune cell activated via the Fc receptor signaling releases lytic enzymes (i.e., granzyme B, perforin) able to induce the apoptosis of the tumor cell [18].

Therefore, HER2-targeted monoclonal antibodies ultimately represent an “unintentional” immunotherapy.

Natural killer (NK) cells, a small population (<10%) of circulating lymphocytes involved in the innate immunity, express Fc-gamma receptors on their surface and can recognize and bind to the reciprocal portion of the antibody (i.e., trastuzumab), which in turn binds to the surface of the target cell (i.e., HER2-positive cancer cell). Therefore, once the Fc receptor binds to the Fc region of trastuzumab, the NK cell releases cytotoxic factors that cause the death of the HER2-positive cancer cell [19].

Since Fc-gamma receptors are key elements involved in ADCC, their polymorphisms were hypothesized to be associated with the magnitude of benefit from trastuzumab and the modification of anti-HER2 monoclonal antibodies’ Fc region has been proposed as a strategy to improve monoclonal antibodies-induced ADCC in patients with HER2-positive breast cancer [20].

Margetuximab is a chimeric monoclonal antibody engineered to increase the affinity for CD16A polymorphisms and decrease affinity for FcγRIIB (CD32B), thus resulting in a more pronounced immune engagement [21]. The SOPHIA trial is a phase III study that compared margetuximab plus chemotherapy vs. trastuzumab plus chemotherapy in 536 heavily pretreated HER2-positive metastatic breast cancer patients, progressing after 2 lines of anti-HER2 treatments. Based on the trial results, margetuximab demonstrated a limited but statistically significant benefit in progression-free survival (PFS) over trastuzumab (median PFS 5.8 vs. 4.9 months, hazard ratio (HR), 0.76; 95% confidence interval (CI), 0.59–0.98; *p* = 0.03), without any advantage in OS (21.6 vs. 19.8 months with margetuximab and with trastuzumab, respectively; HR, 0.89; 95% CI, 0.69–1.13; *p* = 0.33) [22].

Anti-HER2 monoclonal antibodies exert immune-mediated mechanisms of action that involve both innate and adaptive immunity [23,24,25]. Hence, anti-HER2 monoclonal antibodies can trigger a vaccine-like effect in vivo, providing a strong rationale for combination with other immunotherapeutic strategies. Furthermore, the long-term efficacy of dual HER2-blockade strongly supports the substantial immune system contribution to the therapeutic effects of monoclonal antibodies [15,24]. Several ongoing studies are testing anti-HER2 monoclonal antibodies in combination with immunotherapeutic agents that are discussed in the following paragraphs.

## 3. Antibody–Drug Conjugates

Antibody-drug conjugates (ADCs) are monoclonal antibodies biochemically linked to cytotoxic drugs (payload) [26]. The concept behind ADCs is a selective delivery of cytotoxic drugs into tumors that express the monoclonal antibody’s target at higher-than physiologic concentrations. By targeted delivery into cancer cells, ADCs can use payloads that would otherwise be prohibitive if administered outside of this complex and smart structure because of excessive toxicity to normal tissues at therapeutic dosage. The chemical linker between the antibody and the cytotoxic agent is stable in the bloodstream and can be processed in the tumor cell, thus minimizing the systemic exposure to the cytotoxic agent and enhancing the anti-tumor activity [26]. Several ADCs are now available, some already in clinical use and some others in various phases of clinical development.

### 3.1. Trastuzmab-Emtansine (T-DM1)

T-DM1 was the first ADC to be approved for the treatment of HER2-positive breast cancer. It consists of a monoclonal antibody (trastuzumab) linked through a non-cleavable thioether link to a cytotoxic payload (emtansine, a hydrophobic microtubule poison) with a drug–antibody ratio (DAR) of 3.5:1 [27]. The first approval of T-DM1 was based on the results of the EMILIA study. In this large, randomized study T-DM1, compared to capecitabine and lapatinib, showed a significant improvement in survival along with a more favorable toxicity profile in patients with pre-treated metastatic HER2-positive breast cancer [28,29]. The practice-changing results obtained in the metastatic setting prompted the testing of T-DM1 earlier, in the post-neoadjuvant setting, where T-DM1 demonstrated in the KATHERINE trial a significant improvement in invasive disease-free survival (IDFS) in patients with residual disease after the completion of neoadjuvant therapy [30]. So far, trials testing the combination of T-DM1 with other agents (e.g., atezolizumab in KATE2 [31] or pertuzumab in KRISTINE [32]) have failed to show significant improvements in patients treated with T-DM1 in different settings.

### 3.2. Trastuzumab Deruxtecan (T-DXd)

T-DXd is composed of a monoclonal antibody (trastuzumab) combined via a cleavable linker with a cytotoxic payload (deruxtecan, a hydrophilic topoisomerase II inhibitor), with a DAR of 8:1 [33]. These characteristics elicit a potent anti-tumor effect. First, the higher DAR implies a greater quantity of cytotoxic payload delivered into the cancer cell; secondly, T-DXd, differently from T-DM1, can induce the so-called by-stander killing effect: the cytotoxic moiety released by T-DXd inside of the tumor cell is cell-membrane permeable, meaning that can also diffuse to surrounding cells, boosting the anti-tumor activity [33]. This is particularly of note in tumors with heterogeneous or low HER2 expression. Interestingly, T-DXd has so far demonstrated an unprecedented significant efficacy in clinical trials in patients with HER2-positive breast cancer [34,35] and in patients with HER2-low breast cancer [36]. Based on the results of DestinyBreast01, T-DXd has been approved by the Food and Drug Administration (FDA) for the treatment of patients with metastatic HER2-positive breast cancer after two prior lines of therapy [37], paying attention to potentially life-threatening interstitial lung disease as specific drug-related toxicity. Recently, at the ESMO congress 2021, the results of DestinyBreast03 were presented, showing an impressive survival improvement with T-DXd over T-DM1 in patients pre-treated with first-line trastuzumab and taxanes (HR 0.26, 95% CI 0.20–0.35) [34]. Furthermore, a press release confirmed that also Destiny-Breast04 met its primary endpoint, showing improved progression-free survival for patients with hormone receptor positive, HER2-low breast cancer treated with T-DXd compared to chemotherapy. Results will be presented at one of the upcoming medical conferences.

### 3.3. Trastuzumab Duocarmazine (SYD985)

SYD985 is an ADC composed of trastuzumab and duocarmazine, an alkylating agent, as payload (on average 2.8 molecules per monoclonal antibody), each bound to the other via a cleavable linker [38]. In the phase III study TULIP, SYD985 significantly improved PFS compared to chemotherapy in patients with pre-treated HER2-positive metastatic breast cancer (HR 0.64, 95%CI, 0.49–0.84, *p* = 0.002) [39], thus representing a new treatment option for these patients. SYD985 has also been tested in patients with HER2-low breast cancer in a phase I dose-escalation/expansion study, where it showed promising clinical activity and a manageable safety profile [40].

### 3.4. Other ADCs

One of the critical aspects of ADC’s mechanism of action is the efficiency of drug internalization in target cells. One possible strategy to increase this property is to develop antibodies able to recognize two non-overlapping epitopes on the HER2 receptor. This bi-specific antibody structure results in the establishment of HER2 clusters that trigger potent internalization, lysosomal trafficking, and subsequent compound degradation. Accordingly, novel ADCs are being studied, including the XMT-1522, an anti-HER2 antibody conjugated to an auristatin-based cytotoxic payload [41], the MEDI4276, a bispecific, anti-HER2-antibody conjugated to an anti-microtubule agent tubulysin [42] and ZW49, another bi-specific, anti-HER2 ADC targeting the epitopes of trastuzumab and pertuzumab [43]. Actually, the list of so-called “next generation” ADCs is constantly growing, including compounds such as ARX788, A166, BAT8001, and PF-06804103, with clinical results expected in the near future [44].

## 4. Immune-Checkpoint Inhibitors

Adjusted for other immune features, PD-L1 expression is associated with resistance to anti-HER2 monoclonal antibodies, and preclinical studies have suggested that combining trastuzumab with immune checkpoint inhibitors could overcome trastuzumab resistance, thus defining an ideal target for therapy in this context [45].

In metastatic HER2-positive breast cancer, the combination of a PD-1/PD-L1 inhibitor plus an anti-HER2 agent has been first explored in the single-arm, multicenter, phase 1b/2 study PANACEA [46] (Table 1). The PANACEA study enrolled 58 patients with disease progression on prior trastuzumab-based treatment. These patients were treated with pembrolizumab and trastuzumab. The study had two different cohorts: one for PD-L1-positive tumors and one for PD-L1-negative tumors [46]. 77% of the population included in the study had PD-L1-positive disease [46]. An ORR of 15% was observed among patients with PD-L1-positive tumors, while no responses were seen among patients with PD-L1-negative tumors [46]. Subgroup analyses of this study have shown that PD-L1 positive tumors have higher levels of TILs [46].

The KATE2 trial is a multicenter, randomized, double-blind, phase 2 trial that investigated the role of adding atezolizumab to T-DM1, in previously treated metastatic HER2-positive breast cancer [31] (Table 1). According to the study design, 202 patients progressing after treatment with taxane and trastuzumab were randomized to receive either T-DM1 plus atezolizumab or T-DM1 plus placebo, regardless their PD-L1 status [31]. Eventually, the study failed to demonstrate a significant median PFS advantage in the T-DM1 plus atezolizumab arm vs. T-DM1 plus placebo (8.2 vs. 6.8 months respectively; stratified HR 0.82, 95% CI 0.55–1.23; *p* = 0.33) [31]. Although the study did not meet its primary endpoint of PFS in the intention-to-treat (ITT) population, it has been observed a favorable PFS impact of atezolizumab in the subgroup of patients with PD-L1-positive (or with TILs ≥ 5%) tumors, along with a positive trend in terms of OS advantage [31].

Therefore, the use of biomarkers, such as PD-L1 expression and TILs, might help in selecting patients more likely to benefit from immune checkpoint inhibitors in advanced HER2-positive breast cancer [50]. 

Currently, a multicenter, randomized phase 3 trial is evaluating the efficacy of trastuzumab, pertuzumab, and paclitaxel with or without atezolizumab (NCT03199885) as first-line treatment for patients with HER2-positive metastatic breast cancer. The trial aims at randomizing 600 patients with PFS as primary endpoint. 

So far, the role of PD-1/PD-L1 inhibitors in HER2-positive breast cancer has been mainly studied in the metastatic setting. The benefit of such drugs is most evident in first-line therapy as compared to subsequent lines [51,52,53]. The tumor/immune co-evolution leads metastatic breast cancer to be commonly not inflamed. While the disease advances, less immune cells are observed in the tumor microenvironment and less immunogenic antigens are expressed by tumor cells; hence the immune escape progressively augments [54,55,56,57]. Hence, it would be reasonable to anticipate immunotherapy as earlier as possible in the course of the disease, thus moving it to the early stage. In the early stage, neoadjuvant immunotherapy has improved efficacy over adjuvant immunotherapy to eradicate the spread of metastatic disease [58]. Possibly, the different efficacy of immunotherapy in the neoadjuvant setting is at least partially attributable to the use of anthracyclines, which are known to induce immunogenic cell death, thus enhancing the tumor priming phase [59].

In the neoadjuvant setting, two phase-III trials aim at assessing the efficacy of dual HER2 blockade plus PD-L1 inhibition without selecting by PD-L1 status. The IMpassion 050 (NCT03726879) was the first phase III trial to compare the combination of atezolizumab with a neoadjuvant therapy based on dose-dense anthracycline, taxane, trastuzumab, and pertuzumab, vs. the same regimen without atezolizumab, in patients with HER2-positive early breast cancer at high risk (defined as tumor size of more than 2 cm and node-positive disease) [49] (Table 1). Overall, 454 patients were randomized to either treatment arm and, at surgery, those who achieved a pCR continued pertuzumab and trastuzumab, while those with an invasive residual disease could switch to T-DM1 [49]. The study was stopped prematurely due to an unfavorable risk-benefit ratio for patients receiving atezolizumab. Of note, no significant improvement in pCR was observed neither in the ITT population (62.4% with atezolizumab versus 62.7% with placebo, *p* = 1.0), nor in the PD-L1 positive cohort (64.2% with atezolizumab versus 72.5% with placebo, *p* = 0.2) [49].

Table 2 reports the ongoing studies with immune checkpoint inhibitors in HER2-positive breast cancer, that will ultimately help to clarify the role of cancer immunotherapy in early and metastatic HER2-positive breast cancer. The APTneo trial (NCT03595592) is evaluating, in patients with HER2-positive high-risk or locally advanced early breast cancer, the addition—in the neoadjuvant setting—of atezolizumab to the combination of trastuzumab, pertuzumab, carboplatin, and paclitaxel, with the primary endpoint being event-free survival (EFS). After the neoadjuvant part of the study, following surgery, all patients will keep on receiving trastuzumab and pertuzumab for up to one year of anti-HER2 therapy. In addition, patients who received atezolizumab as neoadjuvant therapy will keep on receiving atezolizumab for up to one year. 

Primary endpoints such as IDFS, PFS, and OS may allow a better measure of its immune-mediated anti-tumor effects over time compared to endpoints reflecting tumor shrinkage (e.g., pCR, ORR). In this context, Astefania (NCT04873362) is enrolling patients with HER2-positive early BC and residual disease at surgery following neoadjuvant therapy (Table 2). In this study, 1590 patients will be randomized to either atezolizumab + T-DM1 or placebo + T-DM1. With IDFS as the primary endpoint, the Astefania study will likely be the best study to definitively assess the value of immune checkpoint inhibitors in HER2-positive early breast cancer.

## 5. Chimeric Antigen Receptor (CAR) T Cells

Chimeric antigen receptor (CAR) T cells represent another immunotherapeutic option under investigation for patients with HER2-positive breast cancer. Of note, the CAR strategy combines both the advantages of an antibody-type specificity and the effector function of T-cells, thus bypassing the poor accessibility of HER2-specific antibodies and allowing a potentially broader clinical application in cancer treatment.

HER2-specific CAR T cells have shown some preclinical evidence of anti-tumor activity [60,61].

In vitro, HER2-directed CAR T cells have shown to be able to identify and kill both trastuzumab-sensitive and trastuzumab-resistant cell lines of breast cancer [62].

In vivo, in transgenic mice models, HER2-specific CAR T cells confirmed a consistent anti-tumor activity. Interestingly, the persistence of CAR expression seems to be a crucial element to ensure the success of adoptive cell transfer and killing of tumor cells and, once HER2-specific CAR T cells accumulate at the tumor site, they can proliferate and expand their activity. On the contrary, injection of higher doses of HER2-modified CAR T cells resulted in mice death, alerting on the safety risks associated with high doses [61].

The anti-tumor activity of HER2-specific CAR T cells has also been tested on brain metastases from breast cancer, using orthotopic human tumor xenograft models. HER2-specific CAR T cells were administered locally or loco-regionally, with intra-tumor or intra-ventricular delivery. An enhanced anti-tumor activity was observed, especially if compared to the intravenous delivery of CAR T cells, which resulted in only marginal tumor responses and that required higher doses. Moreover, intracavitary administration could prevent leptomeningeal spread in these models [60].

To further improve understanding and anti-tumor activity of HER2-specific CAR-T cells, additional preclinical, in vitro experiments compared the efficacy of trastuzumab vs. trastuzumab-derived CAR-T cells. Trastuzumab, in the presence of effector NK cells, showed a killing activity restricted to HER2-positive cancer cells on the surface monolayer, while CAR T cells could penetrate the core region of the tumor spheroids and exert a deeper cytotoxic activity. The same experiment conducted on mice showed that treatment with trastuzumab plus effector NK cells could only temporarily delay cancer growth, yet not inducing cancer regression. On the contrary, HER2-specific CAR T cells could eradicate the tumor, with improved long-term survival [63].

CAR T cells are being studied also in combination with other agents. For example, the inhibition of the epithelial-mesenchymal transition (EMT) in cancer cells has been investigated as a possible strategy to enhance the activity of HER2-directed CAR-T cells [64]. Inhibition of the Transforming Growth Factor-beta-1 (TGF-β1) pathway has been shown to block the EMT process in cancer cells in vitro, and, consequently, to restore the cytotoxic activity of HER2-specific CAR-T cells. TGF-β1 inhibitors showed some promising activity in enhancing the killing capacity of HER2-CAR-T cells also in vivo, in a mouse model, and represent an attractive field of investigation [64].

Also PD-1 blockade has been tested in combination with HER2-specific CAR T cells, both in vitro and in xenograft models. Interestingly, the addition of an anti-PD1 antibody demonstrated improving the tumor-killing activity of HER2-specific CAR-T cells, thus representing another strategy worthy of further study [65].

Preclinical data from CAR T cells in HER2-positive breast cancer seem promising, although clinical validation is needed.

In the last few years, CAR-T cells have entered the clinic for patients with hematological malignancies, and, more recently, there has been an increase in clinical trials focusing on CAR-T cells also for patients with solid tumors, especially for cancers of the nervous system [66,67], sarcoma [68] and melanoma [69]. Despite an overall acceptable tolerability, these studies have shown a limited anti-tumor activity so far, which could be due to the immunosuppressive microenvironment and/or to the lack of tumor antigens representing effective therapeutic targets for CAR-T cells. In the specific context of breast cancer, some clinical trials are ongoing and will ultimately shed light on this topic in the next future. Some examples of currently recruiting studies on CAR-T cells in HER2-positive breast cancer include the study NCT03696030, that is testing HER2-CAR-T cells in patients whose cancer has spread to the brain or leptomeninges; the phase I study NCT04650451, testing HER2-Targeted Dual Switch CAR-T Cells (BPX-603) in subjects with HER2-positive solid tumors; the phase 1 study NCT04660929, that is the first-in-human study of CAR macrophages in HER2 overexpressing solid tumors; the phase I study NCT04511871 that is investigating the safety and tolerability of CAR-modified autologous T cells (CCT303-406) in subjects with relapsed or refractory stage IV metastatic HER2-positive solid tumors.

Several open challenges exist in the implementation of CAR-T as a future treatment strategy, including improvement in their safety profile and anti-tumor activity, with a more selective homing to tumors and a more persisting activity [70].

For example, optimal signaling through the intracellular co-stimulatory domains is fundamental to increasing CAR-T cell survival, function and proliferation, and this should be considered in CAR-T cell design [71].

In terms of safety, the potential toxicity of T cells on healthy tissue represents one of the major challenges and potential limitations on CAR-T cell clinical development. Accordingly, some research is investigating how to fine-tune the affinity of CAR-T cells to discriminate between the tumor and the normal tissue, which could express the same targets at physiologic levels [72].

## 6. Vaccines

There are different types of vaccines currently under investigation in HER2-positive breast cancer, including protein-based, cell-based, gene-based, and viral–vector based [73].

Protein-based vaccines are the most widely investigated vaccines, and target constitutive immunogenic peptides of HER2, like AE37 (from the intracellular domain) [74], GP2 (from the transmembrane domain) [75], and E75 (from the extracellular domain) [76].

Nelipepimut-S (NP-S)/NeuVax is an E75-based vaccine that stimulates CD8+ T cytotoxic lymphocytes to recognize and eliminate HER2-expressing cancer cells [77]. Given in combination with an immunoadjuvant (granulocyte macrophage-colony stimulating factor [GM-CSF]), NP-S has been shown to induce E75-specific CD8+ T-cells expansion, which is even greater in patients with HER2-low breast cancer [78] and when NP-S is combined with trastuzumab [79], thus indicating a synergism of this combination. After preliminary studies showing encouraging signs of activity, NP-S has been tested in a phase IIb study, including 275 patients with HER2-low and either node-positive or HR-negative (i.e., TNBC) breast cancer; adjuvant NP-S + GM-CSF and trastuzumab were compared to trastuzumab with GM-CSF alone [76,80]. In the ITT population, no significant difference in DFS was observed (HR 0.62, 95% CI 0.31–1.25, *p* = 0.18), while in an exploratory analysis of TNBC subgroup there was a significant DFS improvement (HR 0.26, 95% CI 0.08–0.81, *p* = 0.01) [76,80]. Similarly, in a randomized, phase III trial including 758 women with node-positive, HER2-low breast cancer in the adjuvant setting, no significant difference in DFS was observed between NP-S and placebo arms (HR 1.56, 95% CI, 0.96–2.55, *p* = 0.07) [81]. While further investigation is ongoing specifically in TNBC, an ongoing trial is testing NP-S/GM-CSF in combination with trastuzumab in patients with high-risk HER2-positive breast cancer in the adjuvant setting (NCT02297698).

GP2 is a subdominant epitope located in the transmembrane domain of HER2. A GP2-based vaccine (654-662, IISAVVGIL) has been tested in a phase IIb study + GM-CSF vs. GM-CSF alone after adjuvant trastuzumab in women with operable breast cancer and expressing any degree of HER2 (1-3+) [75]. After 5 years of follow-up, the estimated DFS rates were 100% in the 46 patients HER2 3+ treated with GP2 + GM-CSF and 89.4% in the 50 HER2 3+ patients treated with GM-CSF alone (*p* = 0.0338) [82]. No difference was observed in patients with HER2 1-2+. A pivotal phase III trial is now testing GP2 in HER2-positive patients in the neoadjuvant setting [82].

AE37 (HER2/Neu 776-790) is a protein-based HER2-directed vaccine designed to target the intracellular domain of HER2. Preclinical studies have shown its ability to stimulate both CD4+ and CD8+ cells in vitro and in vivo [83], providing the rationale for clinical investigation. AE35 failed to show an improvement in DFS in patients with node-positive and high-risk node-negative breast cancer enrolled in a randomized phase II trial, although an exploratory analysis showed a non-significant trend towards benefit in the TNBC subgroup [74]. AE37 is now being tested in combination with pembrolizumab in patients with TNBC (NCT04024800).

Table 2 reports the ongoing trials testing new therapeutic vaccines in HER2-positive breast cancer, including the new multi-epitope HER2 peptide vaccine TPIV100 (NCT04197687).

Cell-based vaccines are mainly patient-specific and are created from a lysate of tumor cells extracted from each specific patient in order to stimulate a personalized immune response against cancer cells. The major limitations of this approach consist in the potential scarce immunogenicity of tumor cells, as well as in the risk of immune-mediated adverse events towards self-antigens included in the lysate [84,85]. This class of vaccines includes autologous cell-based vaccines (e.g., Lapuleucel-T, APC8024), allogenic cell-based vaccines, and dendritic cell-based vaccines. Several phase I studies have tested cell-based vaccines in patients with HER2-positive breast cancer [86,87], demonstrating the safety and feasibility of this approach, and phase II trials are ongoing (Table 2).

In gene-based vaccines, DNA coding for tumor antigens (e.g., HER2) are injected into the host carried by a plasmid. Hence, this type of vaccine stimulates both an antigen-specific and a non-specific innate immune reactions [88]. Again, after promising data from early phase clinical studies [89,90,91], phase II studies are now ongoing. Interestingly, one of them is testing a dendritic cell-based vaccine (DC1) vs. a plasmid-based DNA vaccine (WOKVAC) in patients with residual disease after the completion of neoadjuvant chemotherapy for HER2-positive breast cancer (NCT03384914). Preliminary results are expected in early 2023.

Viral–vector-based vaccines exploit the natural immunogenicity of viruses [92]. In this approach, the genome of viruses can be engineered to carry transgenes of interest; then, once the virus has infected the host cell, these genes coding for tumor antigens can be expressed and become the target of an immune response [92]. An ongoing study (NCT03632941) is evaluating the combination of the viral vector-based vaccine VRP-HER2 and pembrolizumab in patients with advanced HER2-positive breast cancer. As therapeutic vaccines can turn a cold tumor microenvironment into a hot one, the combination with other immunomodulatory therapies (such as immune checkpoint inhibitors) is particularly intriguing.

Overall, although initial trials on therapeutic vaccines in breast cancer showed disappointing results and limited benefit [81,93,94], therapeutic vaccines nowadays represent a promising strategy. Of note, initial trials probably had some important limitations, including a wrong patient selection, that focused the investigation on metastatic, heavily pre-treated (hence immunocompromised) patients, where an active immune reaction could not be reasonably observed. Now, clinical trials are also set up on different patient populations and focus mainly on the adjuvant setting (in some studies also referred to as “prevention of metastases”), where an immune engagement is eventually more predictable.

Moreover, thanks to the advancements in technology boosted by the efforts in the research field due to the SARS-CoV-2 pandemic, new platforms, and new vehicles are being developed, resulting in improved immunogenicity of therapeutic vaccines [73,95].

## 7. Discussion and Future Perspectives

The first generation of “passive immunotherapy” with the use of HER2-directed monoclonal antibodies has significantly improved the outcomes of patients with early and advanced HER2-positive breast cancer. On the other side, while the second wave of immunotherapy proper (especially in the case of immune checkpoint inhibitors) has gained momentum for the treatment of several different solid tumors, it has not performed as well in unselected patients with HER2-positive breast cancer.

Indeed, despite a strong preclinical rationale [45,96] supporting a synergistic effect of immunotherapy and HER2-targeting agents, results from clinical trials have been controversial and rather disappointing so far. Studies exploring the role of immune checkpoint inhibitors in patients with metastatic HER2-positive breast cancer [31,46,47,48] have shown low anti-tumor efficacy in unselected, heavily pretreated patients, with a signal of limited activity restricted to patients with PD-L1-positive tumors [31,46]. Of note, immune response widely differs between the early and the metastatic setting. Early tumors confined to the breast are usually characterized by a more permissive microenvironment, and have a lower degree of tumor evasion, which allows the immune system to “recognize” the tumor antigens and to create an immune response against tumor cells. On the contrary, advanced tumors are characterized by a higher tumor burden and are enriched with resistant cells expressing less immunogenic antigens in the context of an immune-tolerant microenvironment. Additionally, metastatic patients are often systemically immunosuppressed [12,97,98]. Thus, the investigation of immunotherapy in the early setting for patients with HER2-positive breast cancer arose as a more than promising strategy. However, the first large, randomized, phase III trial testing the addition of an immune checkpoint inhibitor (atezolizumab) to dual-anti HER2 blockade and chemotherapy in the neoadjuvant setting is negative [49], and forces to make some considerations about the role of immunotherapy in HER2-positive breast cancer. First, whether these results suggest a lack of anti-tumor activity is controversial. There are indeed several possible reasons behind these negative results: (i) at a trial level, pCR showed a weak association with survival [99], hence findings should be interpreted with caution. In TNBC, the addition of immune checkpoint inhibition to standard neoadjuvant chemotherapy did not improve pCR in the GeparNuevo study, yet inducing a significant increase in EFS (as also confirmed in KEYNOTE-522) [100,101]; (ii) differently from TNBC, HER2-positive breast cancer has a well-established and effective standard of care, represented by dual-anti HER2 blockade, that makes incredibly challenging to identify new treatments able to further improve the clinical outcomes of these patients; (iii) we need to better select patients who can benefit from the addition of immunotherapy, and increasing efforts should be made to identify predictive biomarkers. Clinical trials powered according to survival endpoints (i.e., EFS) as primary endpoints may allow a better measure of immunotherapy’s anti-tumor effects over time, and a better patient selection is warranted instead of running after umpteenth “add-on” study designs.

Several trials are ongoing and will clarify the role of immunotherapy in HER2-positive breast cancer. It is likely that, in the next future, immunotherapy will become part of the treatment landscape for HER2-positive disease, with ADCs and therapeutic vaccines being among the most promising treatment strategies.

ADCs are remarkably changing the treatment landscape of HER2-positive breast cancer [37], and, based on recent promising data from clinical trials [34], an increasingly broader implementation of these agents in clinics is foreseen. Of note, ADCs seem to be active not only in HER2-positive tumors, but also in HER2-low ones [102], thus indicating that a larger proportion of patients could derive benefit from these new drugs. Interestingly, a recent press release disclosed that the DestinyBreast-04 study met its primary endpoint, confirming a significant improvement in PFS for patients with metastatic HER2-low breast cancer treated with T-DXd, compared to chemotherapy of physician’s choice. The study results will be presented at one of the upcoming medical conferences.

Therapeutic vaccines represent another promising treatment strategy and clearly show how remarkable advances in the research field can change the destiny of investigational drugs. Despite initial disappointing results, a better understanding that vaccines can better work in immune-competent subjects, namely in the early rather than advanced setting of disease, together with technology advances triggered by the SARS-CoV-2 pandemic, made this class of agent one of the most promising immunotherapeutic strategies under investigation, so far.

Yet, open challenges exist in the field of immunotherapy for patients with HER2-positive breast cancer, including the identification of predictive biomarkers of response to select patients who may benefit most from these treatments, the implementation of better endpoints to assess their anti-tumor activity in clinical trials, and the management of potential long-term immune-related adverse events, that are not extensively discussed in the present review, but deserve awareness and attention.

## 8. Conclusions

Immunotherapy has revolutionized the treatment and history of several cancer types.

In breast cancer, immune checkpoint inhibitors are already a standard of care in selected patients with a TNBC subtype, both in the early and advanced settings. In HER2-positive breast cancer, a strong preclinical rationale suggests that immunotherapy is an intriguing field, hence, it is under current clinical investigation. Notwithstanding some hurdles, immunotherapy still represents a promising strategy for patients with HER2-positive breast cancer, and ongoing trials will contribute to clarifying its definitive therapeutic role.

## Figures and Tables

**Table 1:** Main studies on immune checkpoint inhibitors in patients with HER2-positive breast cancer.

Study	Phase	Population	Treatment	Main Results
Advanced Setting				
PANACEA [46]	I/II, single arm	HER2+ ABC, progressed to trastuzumab (*n* = 52, PD-L1+ *n* = 40)	Pembrolizumab + trastuzumab	ORR: 15% of PD-L1+ pts No ORs among PD-L1− pts mPFS: 2.7 mos (90% CI 2.6–4.0) in PD-L1+ mPFS: 2.5 mos (90% CI 1.4–2.7) in PD-L1−
KATE-2 [31]	II, randomized	HER2+ ABC, previously treated with trastuzumab and a taxane (*n* = 202, PD-L1+ *n* = 84)	Atezolizumab + T-DM1 vs. Placebo + T-DM1	ORR: 54% vs. 33% in PD-L1+ pts; ORR: 39% vs. 50% in PD-L1− pts mPFS: 8.2 vs. 6.8 mos in ITT (HR 0.82, 95% CI 0.55–1.23) mPFS: 8.5 vs. 4.1 mos in PD-L1+ (HR 0.60, 95% CI 0.32–1.1)
NCT02649686 [47]	I	HER-2 positive ABC, previously treated with trastuzumab and taxanes (*n* = 15, PD-L1+ *n* = 0)	Durvalumab + trastuzumab	ORR: 0/15
JAVELIN Solid Tumors [48]	Ib	ABC refractory to or progressing after standard-of-care therapy (*n* = 26)	Avelumab	ORR: 0/26
Early setting				
IMpassion050 [49]	III	Stage II-III HER2+ EBC (*n* = 454, PD-L1+ *n* = 218)	(ddAC → paclitaxel + pertuzumab + trastuzumab) +/− atezolizumab → surgery → (trastuzumab + pertuzumab) +/− atezolizumab	pCR: 62.4% in atezolizumab arm vs. 62.7% in placebo arm (*p* = 0.9551) in ITT pCR: 64.2% in atezolizumab arm vs. 72.5% in placebo arm (*p* = 0.1846) in PD-L1+ pts

Abbreviations: ABC: advanced breast cancer; OR(R): objective response (rate); PFS: progression-free survival; ITT: intention-to-treat (population); EBC: early breast cancer; ddAC: dose-dense doxorubicin and cyclophosphamide; pCR: pathological complete response.

**Table 2:** Ongoing studies with immune-checkpoint inhibitors (pembrolizumab, nivolumab, atezolizumab, durvalumab, avelumab, and spartalizumab) and other immunotherapies in HER2-positive breast cancer. Data extracted from https://clinicaltrials.gov (accessed on 10 February 2022).

Study (NCT ID)	Phase	Setting	Population	Experimental Treatment	Status
**Pembrolizumab**					
NCT04512261 TOPAZ	I/II	Advanced	Patients with brain metastases	Pembrolizumab + tucatinib + trastuzumab	Suspended (enrollment temporarily on hold pending amendment to the protocol)
NCT04789096 TUGETHER	II	Advanced	Any line, but prior treatment with pembrolizumab and T-DM1 (in any setting) is required	Pembrolizumab + tucatinib + trastuzumab (+/− capecitabine)	Not yet recruiting
NCT03032107	I	Advanced	At least 1 prior line for advanced disease, or PD during or within 6 months from the adjuvant treatment	Pembrolizumab + T-DM1	Active, not recruiting
NCT03841110	I	Advanced	Not available standard treatments	FT500 + immune checkpoint inhibitors (including pembrolizumab) +/− IL2	Recruiting
NCT03632941	II	Advanced	HER2-positive BC, prior pertuzumab + trastuzumab is required	VRP-HER2 vaccination + pembrolizumab	Recruiting
NCT04348747	II	Advanced	Patients with asymptomatic brain metastases	Anti-HER2/HER3 dendritic cell vaccine + pembrolizumab	Not yet recruiting
NCT04042701	I	Advanced	Patients with advanced BC (HER2-positive or HER2-low) or HER2 expressing/mutant NSCLC	Pembrolizumab + trastuzumab deruxtecan	Recruiting
NCT01042379 I-SPY	II	Neoadjuvant	T > 2.5 cm, no prior treatment	Personalized Adaptive novel agents including pembrolizumab	Recruiting
NCT03747120	II	Neoadjuvant	T > 2 cm and/or N+, no prior treatment	Pembrolizumab + trastuzumab + pertuzumab + weekly paclitaxel	Recruiting
NCT03988036 Keyriched-1	II	Neoadjuvant	T1c, N0-N2; T2, N0-N2; T3, N0-N2 with molecular HER2-enriched intrinsic subtype tested by PAM50	Pembrolizumab + trastuzumab + pertuzumab	Recruiting
**Nivolumab**					
NCT03841110	I	Advanced	Not available standard treatments	FT500 + immune checkpoint inhibitors (including nivolumab) +/− IL2	Recruiting
NCT03523572	I	Advanced	Advanced BC (HER2-positive and HER2-low) and urothelial Cancer	Nivolumab + trastuzumab deruxtecan	Active, not recruiting
**Atezolizumab**					
NCT03125928	II	Advanced	1st line	Atezolizumab + paclitaxel + trastuzumab + pertuzumab	Recruiting
NCT03650348	I	Advanced	Any line	PRS-343 + atezolizumab	Active, not recruiting
NCT03417544	II	Advanced	Patients with brain metastases	atezolizumab + pertuzumab + trastuzumab	Active, not recruiting
NCT04759248 ATREZZO	II	Advanced	Any line, prior pertuzumab/trastuzumab and T-DM1 is required	Atezolizumab + trastuzumab + vinorelbine	Recruiting
NCT03841110	I	Advanced	Not available standard treatments	FT500 + immune checkpoint inhibitors (including atezolizumab) +/− IL2	Recruiting
NCT03199885 NRG BR004	III	Advanced	1st line	Atezolizumab + taxane + trastuzumab + pertuzumab	Recruiting
NCT04740918 KATE3	III	Advanced	Prior Trastuzumab- (+/− Pertuzumab) and Taxane-Based Therapy is required	Atezolizumab + T-DM1	Recruiting
NCT03726879 IMpassion050	III	Neoadjuvant	T2-T4, N1-N3, M0	Atezolizumab + doxorubicin + cyclophosphamide → paclitaxel + trastuzumab + pertuzumab	Active, not recruiting
NCT03881878	I/II	(Neo)adjuvant	T2-3N0-3 or T1cN1	Atezolizumab + docetaxel + trastuzumab + pertuzumab → surgery → adjuvant atezolizumab + trastuzumab + pertuzumab (+ doxorubicine and cyclophosphamide for non-pCR patients)	Not yet recruiting
NCT03595592 APTneo	III	(Neo)adjuvant	T1cN1, T2N1, T3N0, or locally advanced and inflammatory breast cancers	Atezolizumab + trastuzumab + pertuzumab + carboplatin + paclitaxel → surgery → adjuvant atezolizumab + trastuzumab + pertuzumab	Recruiting
NCT04873362 Astefania	III	Adjuvant	cT4/anyN/M0, any cT/N2-3/M0 or cT1-3/N0-1/M0 at presentation, treated with neoadjuvant therapy and surgery	Atezolizumab + T-DM1	Recruiting
**Avelumab**					
NCT03414658 AVIATOR	II	Advanced	Prior T-DM1 (in any setting) and prior pertuzumab and trastuzumab are required	Avelumab + trastuzumab +/− vinorelbine +/− utomilumab	Recruiting
**Durvalumab**					
NCT04538742 DB-07	I/II	Advanced	≥ 2nd line (phase I); ≥ 1st line (phase II)	Trastuzumab deruxtecan + durvalumab + paclitaxel	Recruiting
NCT01042379 I-SPY	II	Neoadjuvant	T > 2.5 cm, no prior treatment	Personalized adaptive novel agents including durvalumab	Recruiting
**Spartalizumab**					
NCT04802876 ACROPOLI	II	Advanced	PD1-high mRNA expressing solid tumors	Spartalizumab	Recruiting
**Other immunotherapies**					
**HER2-CAR-T**					
NCT01219907	I	Advanced	Any line	Ex vivo-expanded HER2-specific T cells and cyclophosphamide after vaccine therapy	Withdrawn
NCT03696030	I	Advanced	Patients with brain or leptomeningeal metastases	Chimeric antigen receptor (CAR) T-cell therapy	Recruiting
NCT04660929	I	Advanced	No available standard treatment options	CAR-macrophages (CT-0508)	Recruiting
NCT03319459	I	Advanced	Solid tumors including HER2-positive breast cancer	FATE-NK100 + trastuzumab	Completed
NCT02843126	I/II	Advanced	No available standard treatment options	Trastuzumab + NK immunotherapy	Completed
NCT04650451	I	Advanced	Subjects with HER2-positive solid tumors	HER2-targeted dual-switch CAR-T cells (BPX-603)	Recruiting
NCT04511871	I	Advanced	Patients with relapsed or refractory HER2 positive solid tumors	Autologous T cell modified chimeric antigen receptor (CAR) (CCT303-406)	Recruiting
NCT02491697	II	Advanced	Stage IV BC (any subtype)	DC-CIK immunotherapy + capecitabine	Active, not recruiting
**Vaccines**					
NCT00194714	I/II	Advanced	Stable disease on trastuzumab monotherapy	HER-2/neu peptide vaccine + trastuzumab	Active, not recruiting
NCT00436254	I	Advanced	Stage III–IV HER2-positive breast cancer with metastasis in remission	DNA plasmid-based vaccine encoding the HER-2/Neu Intracellular domain + GM-CSF	Active, not recruiting
NCT02297698	II	Adjuvant	HER2-positive BC at high risk of relapse	NeuVax vaccine (nelipepimut-S/GM-CSF) + trastuzumab	Active, not recruiting
NCT00393783	I	Advanced	Stage III–IV HER2- positive BC	Xenogeneic HER2/Neu DNA immunization	Active, not recruiting
NCT04521764	I	Advanced	Stage IV BC (any subtype)	Oncolytic measles virus Encoding Helicobacter pylori neutrophil-activating protein (MV-s-NAP) vaccine	Recruiting
NCT01376505	I	Advanced	Solid tumors including BC	MVF-HER-2 Vaccine	Recruiting
NCT03328026	I/II	Advanced	Stage IV BC (any subtype)	SV-BR-1-GM in combination With INCMGA00012 and epacadostat	Recruiting
NCT04246671	I/II	Advanced	Stage IV HER2-positive BC	Intravenous TAEK-VAC-HerBy vaccine	Recruiting
NCT03630809	II	Early and advanced	Patients with metastatic and early HER2-positive breast cancer	HER2-pulsed DC1 vaccine	Suspended for protocol revision
NCT03387553	I	Neoadjuvant	Patients candidate to receive neoadjuvant therapy	HER2 directed dendritic cell vaccine + neoadjuvant standard therapy	Active, not recruiting
NCT04329065	II	Neoadjuvant	Stage I–III ER-negative/HER2- positive BC	WOKVAC vaccination (pUMVC3-IGFBP2-HER2-IGF1R plasmid DNA vaccine) + pertuzumab + trastuzumab + paclitaxel	Recruiting
NCT04197687	II	Post-neoadjuvant	Stage II/III in patients with residual disease after chemotherapy and surgery	T-DM1 + TPIV100 (multi-epitope HER2 peptide vaccine) + GM-CSF	Recruiting
NCT02061423	I	Post-neoadjuvant	Stage I–III HER2-positive BC with residual disease post-neoadjuvant CT	HER-2 pulsed dendritic cell vaccine	Active, not recruiting
NCT03384914	II	Adjuvant	Residual invasive disease after neoadjuvant therapy	DC1 vaccine vs. WOKVAC vaccine	Recruiting
**Others**					
NCT00684983	II	Advanced	Prior treatment with trastuzumab and anthracycline or taxane is required	Capecitabine + lapatinib ditosylate +/− cixutumumab	Completed
NCT04120246	I	Advanced	Any line	Alpha-tocopheryloxyacetic acid (TEA) + trastuzumab	Recruiting
NCT04307329 MIMOSA	II	Advanced	At least one and maximum 3 prior lines of palliative chemotherapy	Monalizumab + trastuzumab	Recruiting
NCT03571633 BREASTIMMU02	II	Neoadjuvant	T > 20 mm, cN0 or cN1, M0, previously treated with 4 cycles of standard adriamycine/cyclophosphamide	Pegfilgrastim + trastuzumab + paclitaxel → surgery → adjuvant trastuzumab (+/− endocrine therapy)	Recruiting
NCT03620201	I	Neoadjuvant	Stage II–III	M7824 (Bintrafusp Alfa) + neoadjuvant standard therapy	Recruiting
NCT01042379 I-SPY	II	Neoadjuvant	T > 2.5 cm, no prior treatment	Personalized adaptive novel agents including immunotherapy	Recruiting

Abbreviations: PD: progressive disease; T: tumor size; N: nodal status; pCR: pathological complete response; BC: breast cancer.
